# Colonic Metastasis of Lung Adenocarcinoma: A Case Report

**DOI:** 10.7759/cureus.4341

**Published:** 2019-03-28

**Authors:** Nathaniel Parker, Chloe McBride, John Forge, Daniel Lalich

**Affiliations:** 1 Internal Medicine, University of Kansas School of Medicine, Wichita, USA; 2 Pathology, Wesley Medical Center, Wichita, USA

**Keywords:** colonic metastasis, primary lung cancer, non-small cell lung cancer, lung adenocarcinoma, rare

## Abstract

Colonic metastases are extremely rare, regardless of primary lung cancer type. A 64-year-old female was referred to the hospital by her gastroenterologist after a same-day colonoscopy revealed a large rectosigmoid mass resulting in near total rectal occlusion. On admission, she complained of abdominal pain and constipation. She had a past medical history of non-small cell lung cancer (T1bN3M0 stage IIIB), diagnosed one year prior. She was thought to be in remission following radiation and immunotherapy with pembrolizumab. She underwent urgent surgical intervention and mass resection with tissue sampling. Pathology confirmed the diagnosis of metastatic lung adenocarcinoma. Systemic chemotherapy with pemetrexed and carboplatin followed by localized radiation to the pelvic region was administered. A refractory pelvic region tumor growth was evident on subsequent imaging. Cessation of chemoradiation therapy occurred after the patient experienced a debilitating stroke and she was transferred to hospice care. Colonic metastasis should be considered when patients with a history of primary lung cancer have abdominal symptoms.

## Introduction

Lung cancer is the most common cause of cancer-related deaths in the United States and worldwide [1-2]. Non-small cell lung carcinoma (NSCLC) accounts for approximately 80% of all lung cancer diagnoses [1-2]. Although almost 50% of NSCLC patients present with metastatic disease at the time of diagnosis, colonic metastases are rare, regardless of primary lung cancer type [3-4]. Autopsy studies suggest the incidence of asymptomatic colonic metastasis is approximately 12% [5-10]. Symptomatic colonic metastasis is even rarer [5-6,8,11-14]. This report describes a rare case of colonic metastasis from primary lung adenocarcinoma.

## Case presentation

A 64-year-old female was referred to the hospital by her gastroenterologist after a same-day colonoscopy revealed a large rectosigmoid mass resulting in near total rectal occlusion. She had a past medical history of tobacco smoking and NSCLC (T1bN3M0 stage IIIB) of the adenocarcinomatous type, diagnosed one year prior. She was thought to be in remission following radiation and immunotherapy with pembrolizumab.

On admission, she complained of progressively worsening right upper quadrant abdominal pain and constipation. Vital signs, physical examination, and laboratory testing were primarily benign. Computerized tomography (CT) imaging showed a severe colonic stool burden and a single soft tissue left upper lobe lung mass consistent with the patient’s NSCLC history. Also, a large soft tissue mass with mucosal invasion in the rectosigmoid colon was evident. She underwent an urgent colostomy, ileocecectomy, anastomosis, and rectosigmoid mass resection with tissue sampling. Histopathology favored poorly differentiated adenocarcinoma. H&E staining showed extensive necrosis, focal mucosal involvement, and negativity for regional lymph node carcinoma. Properly controlled immunohistochemical (IHC) staining was performed, which revealed a strong positive immunoreactivity for CK7 and positive TTF-1 Napsin-A, Moc-31, and Ber-EP4. Only minimal focal staining for p63, CK5, and CK6 was observed. The tumor tissue was negative for CDX2, CK20, CD45, MART-1, GCDFP-15, ER, synaptophysin, NCAM/CD56, and chromogranin. Mucicarmine staining was equivocal for intracytoplasmic mucin. This IHC staining profile (strongly positive CK7 and positive TTF-1/Napsin-A with negative CDX2/CK20) supported metastatic adenocarcinoma of lung origin, rather than primary colorectal adenocarcinoma. Her postoperative course was uneventful, and she was discharged after the operation.

The patient was started on systemic chemotherapy with carboplatin and pemetrexed followed by radiation to the pelvic region. After a few treatment cycles, she developed considerable pelvic pain, resulting in a significant performance status decline and experienced multiple infections requiring hospitalizations. Subsequent positron emission tomography (PET)-CT scans suggested refractory pelvic tumor growth. Additional radiation for the palliation of pain by reducing the pelvic tumor size was determined as reasonable. However, the patient experienced a debilitating stroke and was transferred to hospice care.

## Discussion

The most common metastatic site of NSCLC is bone (34%), followed by lungs (32%) and brain (28%) [15]. Colonic metastasis is uncommon with an incidence of 0.1% [16]. The small bowel serves as the most common site of metastatic gastrointestinal involvement [5]. This could be due to the enhanced potential of small bowel malignancies to cause serious complications, such as perforation, obstruction, or bleeding [5-6]. Histological examination remains the gold standard for diagnosis [10,17]. Average survival following the discovery of colonic metastasis to death has been reported to be approximately two months [5,10]. Early detection and surgical intervention have been postulated to improve survival [17].

## Conclusions

This report presented a unique and rare case of colonic metastasis of primary lung adenocarcinoma. Patients with a history of primary lung cancer that present with abdominal symptoms may have bowel metastasis from lung cancer. Thus, expedited intestinal tract investigation should be done to allow for early detection and treatment. More reports on the colonic metastasis of lung carcinoma are required to clarify clinical features and outcomes.
